# Simulation of Thermal Behavior of Glass Fiber/Phenolic Composites Exposed to Heat Flux on One Side

**DOI:** 10.3390/ma13020421

**Published:** 2020-01-16

**Authors:** Han Li, Nasidan Wang, Xuefei Han, Baoxin Fan, Zhenyu Feng, Shijun Guo

**Affiliations:** 1 School of Mechatronical Engineering, Beijing Institute of Technology, Beijing 100081, China; s.guo@cranfield.ac.uk; 2 College of Airworthiness, Civil Aviation University of China, Tianjin 300300, China; nsdwang@163.com (N.W.); hanxuefei185@163.com (X.H.); fan1120120241@163.com (B.F.); mhfzy@163.com (Z.F.); 3 Centre of Aeronautics, Cranfield University, Bedfordshire MK43 0AL, UK

**Keywords:** thermal behavior, glass fiber/phenolic composite, decomposition, finite element analysis

## Abstract

A 3D thermal response model is developed to evaluate the thermal behavior of glass fiber/phenolic composite exposed to heat flux on one side. The model is built upon heat transfer and energy conservation equations in which the heat transfer is in the form of anisotropic heat conduction, absorption by matrix decomposition, and diffusion of gas. Arrhenius equation is used to characterize the pyrolysis reaction of the materials. The diffusion equation for the decomposition gas is included for mass conservation. The temperature, density, decomposition degree, and rate are extracted to analyze the process of material decomposition, which is implemented by using the UMATHT (User subroutine to define a material’s thermal behavior) and USDFLD (User subroutine to redefine field variables) subroutines via ABAQUS code. By comparing the analysis results with experimental data, it is found that the model is valid to simulate the evolution of a glass fiber/phenolic composite exposed to heat flux from one side. The comparison also shows that longer time is taken to complete the pyrolysis reaction with increasing depth for materials from the numerical simulation, and the char region and the pyrolysis reaction region enlarge further with increasing time. Furthermore, the decomposition degree and temperature are correlated with depths, as well as the peak value of decomposition rate and the time to reach the peak value.

## 1. Introduction

Recently, fiber reinforced polymer (FRP) composites have been significantly used to construct components of airplane, such as fuselage, wing, empennage, and other structures [1,2] because they have many excellent characteristics, for instance lightweight, high specific intensity, good corrosion and fatigue performance [3,4]. Nonetheless, fire is one of the major security threats in the application of composites. For example, in July 2013, a Boeing 787 airliner caught fire at Heathrow airport in London, which was due to the failure of ELT device close the tail of the fuselage, leading to the skin and frame of carbon fiber epoxy composite fuselage suffering serious thermal damage with significant resin loss and soot deposits [5]. In the airworthiness by FAA, there is a requirement to ensure the safety of using composite materials in civil airframe. For instance, AC 20-107B [6] indicates that the flame retardant and fire-resistant requirements should be taken into account in the design of composite structures, especially exposure to flame exceeding maximum operating temperature. It is therefore essential to develop a numerical model of composite thermal response in order to simulate the thermal behavior of composite in fire.

Many researchers have worked on predicting the thermal response of polymer composites decomposing at high temperatures using different mathematical models. Kung et al. [7] established a thermochemical model of wood burning process, which can be used to analyze the convection process of transient heat conduction, pyrolysis, and volatile gases. Henderson et al. [8] proposed a 1D fugacious thermal model for composites with measured temperature-related thermo-physical parameters and reaction kinetic parameters which are used as inputs to the model in consideration of matrix pyrolysis and gas diffusion in the depth orientation. Then, the temperature distribution of the glass fiber/phenolic resin composite under one-sided heating was predicted, and the results were very consistent with the measured results. Furthermore, Henderson and Wiecek [9,10] improved the 1D thermal model to predict the thermal behavior of the composite material, taking into account the thermochemical expansion [11] in the depth orientation of the sample, as well as the flow and accumulation of decomposition gases. Gibson and Mouritz et al. [12,13,14,15,16], Kandare [17], Desjardin [18], Summers et al. [19], and Bhat et al. [20,21] further improved the pyrolysis kinetic model for thermal analysis of composite laminates in a fire environment. Anjang et al. [22] developed a thermal model for sandwich composites by improving the Henderson and Gibson models. Mike and Vizzini [23] established a three-dimensional thermal model of composites based on Henderson’s one-dimensional transient thermal model, taking into account the accumulation of material energy and anisotropic heat transfer for predicting the temperature distribution of carbon fiber/epoxy resin composite exposed to heat flow of 17.6 kW/m^2^. Florio et al. [24] established a thermal model of ablative glass-filled composites with regard to matrix decomposition, expansion, and the heat exchange caused by the thermal imbalance between the decomposition gas and the solid material. In 2009, Mouritz et al. [25] reviewed the research progress of structural modeling of composite materials under fire, and summarized the analytical models of thermal, chemical, physical and failure procedures affecting the structural response of materials in fire. Yang et al. [26,27] established a finite element model for ablation temperature field calculation under moving boundary conditions for carbon/carbon composites, in which the internal pyrolysis behavior of materials was presented by Arrhenius law [28]. In order to study the ablation behavior of carbon/phenolic composites, Zhu et al. [29] established a mathematical model to predict the surface temperature and the depth of the carbonized region of the material.

With the rapid development of modern design and analysis tools, commercial finite element software is used more and more widely to simulate the thermal behavior of compound materials in fire. Shi et al. [30] predicted the thermo-mechanical characteristics of a silica/phenolic compound material, and investigated spatially dependent temperature and orifice pressure, displacement, and stress contours using COMSOL-Multiphysics commercial finite element software for the coupled temperature-proliferation-deformation problem. Zhang [31] developed a 3D model including the influence of orthotropic viscoelasticity and pyrolysis using commercial finite element software ABAQUS, in order to forecast the thermomechanical characteristics and compressive failure of polymer matrix composites (PMCs) subjected to high temperature and compressive loading. Rizk et al. [32] exploited a 3D thermal model for sandwich faceplates with glass/polyester skins and balsa core, defining the thermal behavior of materials using the UMATHT (User subroutine to define a material’s thermal behavior ) subroutine, in order to forecast the development of the temperature gradient traverse a sandwich compound material structure when exposed to high temperature. Luo et al. [33,34,35,36] established a thermomechanical damage model and developed a finite element method using UMATHT and UMAT in ABAQUS, so as to resolve the thermal and mechanic equations for glass/phenolic compound materials withstanding high temperature and thermal radiative surroundings in consideration of the carbonization of sandwich composites, the decomposition of resins, the reduction of elastic modulus, and the delamination of panels and cores. Pauline et al. [37,38,39] developed a 3D thermochemical model using SAMCEF software to forecast the temperature contour, the mass loss and the pyrolysis front of a carbon fiber epoxy resin compound laminate (T700/M21 compound material) exposed to high temperature.

Glass fiber/phenolic composite is commonly used in the interior of civil airplane due to its better flame retardant performance [40,41,42]. In the incidence of fire or high temperature, the rearrangement of bonds will take place inside of the material, which not only promotes the formation of char, but also reduces the delivery of combustible hydrogen carbide to the flame area. In addition, it is easy for the phenolic resin to carbonize and form a covering char layer and have good flame retardancy. Although the temperature profile of glass fiber/phenolic composite has been predicted and verified in literature [8], the carbonization process and the characterization and regularity of the decomposition of glass fiber/phenolic composite is yet to be simulated and evaluated.

In the present study, a transient thermal response model for glass fiber/phenolic composite is developed to represent the thermal characteristics (UMATHT) and the procedure of decomposition comprehensively. Firstly, heat transfer equation, decomposition rate equation, and continuity equation are applied in the model to depict the thermal behavior of glass fiber/phenolic composite. The anisotropic heat conduction, pyrolysis gas convection and matrix pyrolysis of the material have also been considered. Secondly, the model is implemented by using an ABAQUS based finite element method interfacing with user defined subroutine code. The FE model of cylindrical glass fiber/phenolic composite exposed to heat flux on one side was established using the thermophysical and geometrical parameters of material and boundary condition given in reference [8]. The temperature profiles are computed in order to prove the validity of the model by comparing the predicted results and the experimental data. Lastly, the decomposition degree and the rate of decomposition of the material are calculated by updating the density and redefining the field variables (USDFLD) in time, and the changes of thermophysical properties in the process of decomposition are extracted and discussed. The model plays an important role to gain insight of the thermal characteristics of glass fiber/phenolic composite exposed to one-sided heat flux.

## 2. Theoretical Model

### 2.1. Three-Dimensional Heat Transfer

Heat transfer equation for composite materials in the case of unidirectional flow of gases is introduced as Equation (1), in accordance with the following hypothesis [10]:

1. No accumulation of pyrolysis gas in the solid composite,
2. No thermo-chemical and volumetric expansion,
3. Thermal balance between the pyrolysis gases and solid composites.

(1) $$\frac{\partial }{\partial t}\left(\rho h_{s}\right)-\nabla •\left(k_{1}\frac{\partial T}{\partial x}i+k_{2}\frac{\partial T}{\partial y}j+k_{3}\frac{\partial T}{\partial z}k\right)+\frac{\partial }{\partial z}\left({\dot{m}}_{g}^{\prime }h_{g}\right)+Q\frac{\partial \rho }{\partial t}=0,$$

where $k_{i}(i=1,2,3)$ are thermal conductivities of composites in three different directions; T, t, ρ, ${\dot{m}}_{g}^{\prime }$ are temperature, time, solid density, mass flux of gases, respectively; $C_{p}$, $C_{pg}$ are solid specific heat, specific heat of gases produced from pyrolysis of resin, separately; $h_{s}=\int_{T_{0}}^{T}C_{p}dT$, $h_{g}=\int_{T_{0}}^{T}C_{pg}dT$ are solid enthalpy and enthalpy of gases, respectively; Q is decomposition heat. The first term indicates the proportion of internal energy change per individual bulk. The second item shows the heat conduction. The thermal conductivity in the three mutually perpendicular directions, $k_{i}(i=1,2,3)$, included in this item is a function of both temperature and the level of pyrolysis of the material. The convection of energy resulting from the gaseous products flowing back through the char structure is given by the tertiary item. The rate of heat generation or consumption resulting from the decomposition is represented by the final term. Definitely, the rate of application of this energy is affected by the rate of pyrolysis. If the pyrolysis procedure is endothermic, the heat of pyrolysis is negative; otherwise, the heat of decomposition is positive. The pyrolysis reaction of the phenolic resin herein is endothermic.

### 2.2. Modeling Decomposition

The Arrhenius equation can draw the influence of temperature on the rate of pyrolysis reaction [43]. In this paper, only consider pyrolysis of resin, which is designated by:

(2) $$\frac{\partial \rho }{\partial t}=-A\left(\rho_{v}-\rho_{d}\right){\left[\frac{\rho -\rho_{d}}{\rho_{v}-\rho_{d}}\right]}^{n}\cdot e^{-E/RT},$$

where $\rho ,\;\rho_{v},\;\rho_{d}$ are the momentary density, the origin density, and the decomposed density, separately; R=8.314 (J/gmol×K) is universal gas constant. *A* is pre-exponential factor, *E* is activation energy, and *n* is the order of pyrolysis reaction. These parameters are essential to describe the pyrolysis process. The Arrhenius parameters *A*, *n*, and *E* can be calculated from thermos-gravimetric data using a modified version of Friedman’s multiple heating rate technique.

If we ignore the accumulation of gases, and consider only flows in the thickness direction, the conservation of mass represents:

(3) $$\frac{\partial {\dot{m}}_{g}^{\prime }}{\partial z}=-\frac{\partial \rho }{\partial t}.$$

### 2.3. Thermal Performances at Distinct Material Statuses

Temperature and the decomposition state of the material influence thermal conductivity and specific heat capacity. At multiple of material statuses—origin, decomposing, and decomposed material—material have distinct thermal performances. Thermal performances at origin and decomposed states may be defined by thermal experiments—for instance, DSC and LFA. Rule of mixture [43] can calculate thermal performances at decomposing status, given as follows:

(4) $$F=\left(\rho -\rho_{d}\right)/\left(\rho_{v}-\rho_{d}\right),$$

(5) $$k_{i}=Fk_{vi}+(1-F)k_{di}\;(i=1,2,3),$$

(6) $$C=FC_{v}+(1-F)C_{d},$$

where $k_{vi},\;k_{di}\;(i=1,2,3)$ are thermal conductivities of virgin and decomposed material in three coordinate directions. $C_{v},\;C_{d}$ are specific heat capacities at origin and decomposed statuses. *F* is remaining mass fractional number of material to virgin material, while decomposition degree equals one minus *F*.

### 2.4. Thermal Boundary Conditions

For the purpose of numerically reproducing the trial enactment, a heat flux has been used as border restriction to the impacted front surface, as illustrated in Equation (7):

(7) $$q_{S,0}^{\prime \prime }=\left(q_{\mathrm{rad}}^{\prime \prime }-\varepsilon_{S}\sigma T_{S}^{4}\right)+h_{\mathrm{conv}}\left(T_{\infty }-T_{S}\right).$$

The border restriction corresponded to the unexposed surface of the composite is illustrated in Equation (8):

(8) $$q_{S,L}^{\prime \prime }=h_{\mathrm{rear}}\left(T_{\infty }-T_{\mathrm{rear}}\right)+\sigma \varepsilon_{S}\left(T_{\infty }^{4}-T_{\mathrm{rear}}^{4}\right).$$

Equations (7) and (8) have considered the radiation and convective heat changes with the circumstance. In the boundary equations above, $q_{\mathrm{rad}}^{\prime \prime }$ represents the radiant heat flux. $\varepsilon_{S}$ is emissivity of the impacted front surface. σ is the Stefan–Boltzmann Constant. $T_{S}$ is the temperature of the impacted front surface. $T_{\infty }$ is the temperature of circumstance. $T_{\mathrm{rear}}$ is the temperature of the unexposed surface. $h_{\mathrm{conv}}$ is the convection heat transfer modulus of the impacted front surface. $h_{\mathrm{rear}}$ is the convection heat transfer modulus of the unexposed surface.

## 3. Experiment and Numerical Simulation Method

### 3.1. Experimental Setup and Measurement

The test sample is a solid rod of 10 mm diameter and 30 mm length made of 39.5% phenolic resin and 60.5% chopped fiber. To measure the temperature in the experiment, four holes of 0.37 mm diameter were drilled through the front surface center of the sample in depth of 1, 5, 10, and 29 mm. The sample was counter drilled with 0.66 mm diameter holes to accommodate the thermocouple junctions. The chromel-alumel thermocouples of 0.254 mm diameter were inserted in the sample through the holes, which were then filled with powder of the same material. After installing the thermocouples, the samples were covered with an alumina insulating jacket as an insulated boundary.

**Figure 1:**
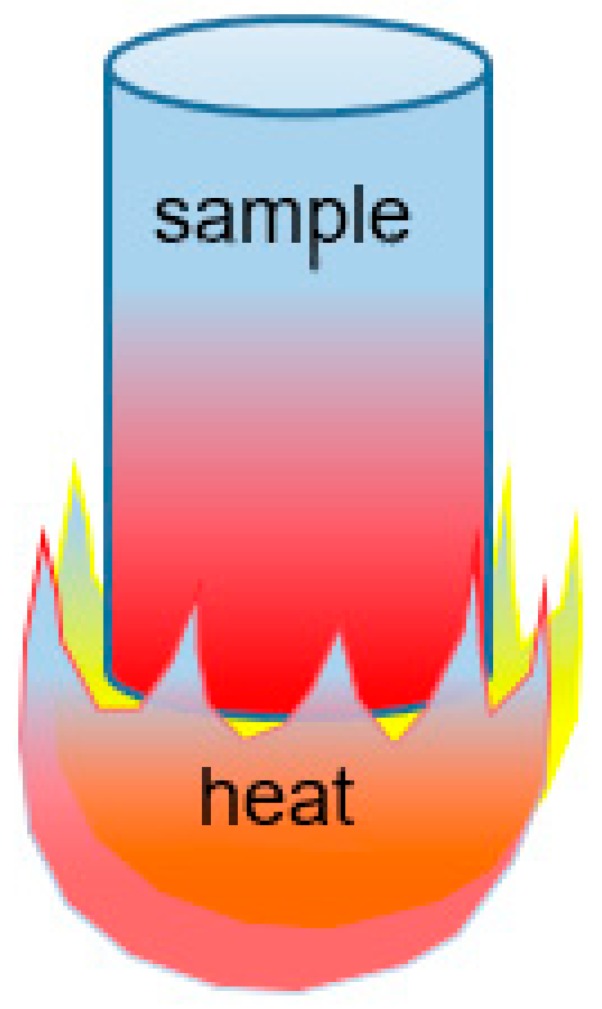
Sample with heat flux at one end.

An experimental apparatus [44] was used to measure the in-depth, time-dependent temperature profiles of the samples exposed to a radiant heat flux as illustrated in Figure 1. The experimental apparatus consists of a silicon carbide heating element mounted vertically below a water-cooled aluminum base plate and a sample carriage mechanism mounted above the base plate. The sample was held vertically by the carriage mechanism above the heating element, which was centered over the heating element and held above the carriage plate prior to testing. It was noted that the sample was held vertically only 1 mm above the heating element during the test. This is an important feature since an increased distance between the test sample and the heat flux would have a significant effect on the results. After preparation, the unit was sealed, purged with argon, and the heating element was raised to the desired temperature. Once the desired heat flux was attained, the test was initiated and conducted until the sample temperatures approached steady-state. It should be noted that the decomposition gas was convected away from the upper surface of the sample by the purge gas flowing from the heating element tube. As a result, a flow field of approximately one dimension inside the sample was created in the test.

### 3.2. Finite Element Model and Implementation

**Figure 2:**
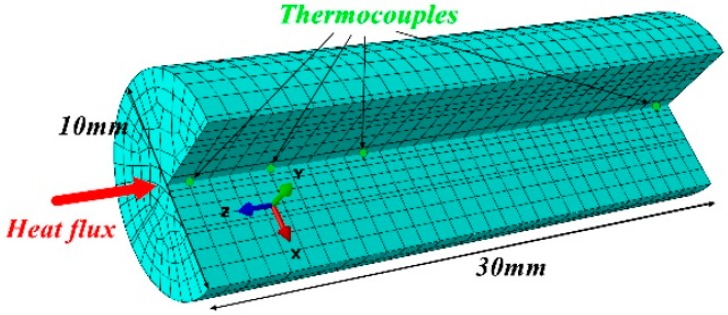
Finite element model of the test sample.

The FE method was used to model the thermal behavior of chopped glass fiber phenolic composite, whose performance was given in Table 1. The FE model was established using DC3D8 unit with a *z*-direction in the longitudinal direction of the sample. The FE model mesh density is a total of 3780 number of elements as shown in Figure 2. A uniform heat flow of 279.7 kW/m^2^ was applied to the front surface of the sample to represent a severe fire environment for a duration of 800 s. The material parameters in the literature [8] are used in the model to calculate the temperature, decomposition degree, and rate. The experimental results in the literature are used to compare and validate the model.

The following parameters were used and updated in the model subroutine UMATHT: the heat flux vector f and variation with temperature and spatial gradients of temperature; internal energy per unit mass U and variation with temperature and spatial gradients of temperature; the solution-dependent state variables at the end of increment updated to their values. The components of the heat flux and spatial gradients are in directions that depend on the local orientations. The decomposition equation is implemented in heat transfer UMATHT using finite forward difference to update the remaining density of material.

Equation (1) is rewritten by expanding the first item using divergence regulations and integrating the heat convection item applying Equation (3), obtaining the ultimate modus of heat transfer equation for finite element fulfillment:

(9) $$\rho C_{p}\frac{\partial T}{\partial t}-\nabla •\left(k_{1}\frac{\partial T}{\partial x}i+k_{2}\frac{\partial T}{\partial y}j+k_{3}\frac{\partial T}{\partial z}k\right)+{\dot{m}}_{g}^{\prime }C_{pg}\frac{\partial T}{\partial z}+\left(h_{s}+Q-h_{g}\right)\frac{\partial \rho }{\partial t}=0.$$

The cardinal energy equilibrium for heat transfer analysis is defined by:

(10) $$\int_{V}\rho \dot{U}dV=\int_{S}qdS+\int_{V}rdV,$$

where V is the bulk of the solid material with surface domain S, ρ is the density of the material, $\dot{U}$ is the material time proportion of the internal energy, q is the heat flux per individual domain of the body spreading into the body, and r is the heat supplied externally into the body per individual bulk.

A heat flux vector f is given such that:

(11) q=−f•n,

where n is the unit vector outward normal to the surface S. Introducing the above relation into the energy balance equation and using the difference law, the energy equilibrium equation can be rewritten as:

(12) $$\int_{V}\rho \dot{U}dV=-\int_{V}\frac{\partial }{\partial x}•fdV+\int_{V}rdV.$$

The internal energy U, the heat flux vector f, and their partial derivatives with respect to temperature and temperature dimensional gradients are the interface variables provided by UMATHT for numerical fulfillment [45]. These variables must be updated to their values at the finish of every time increment.

Combining Equation (9) with Equation (12) yields the time rate of the internal thermal energy and the heat flux vector:

(13) $$\dot{U}=C_{p}\frac{\partial T}{\partial t}+\frac{1}{\rho }\left(Q_{i}+h_{s}-h_{g}\right)\frac{\partial \rho }{\partial t}+\frac{C_{pg}}{\rho }\int_{l}^{z}\frac{\partial \rho }{\partial t}dz\frac{\partial T}{\partial z},$$

(14) $$f=-k_{1}\frac{\partial T}{\partial x}i-k_{2}\frac{\partial T}{\partial y}j-k_{3}\frac{\partial T}{\partial z}k.$$

Applying the finite difference approximation, the incremental modus of Equation (13) is showed as:

(15) $$\Delta U=C_{p}\Delta T+\left(Q_{i}+h_{s}-h_{g}\right)\frac{\Delta \rho }{\rho }+\frac{C_{pg}}{\rho }\int_{l}^{z}\frac{\partial \rho }{\partial t}dz\frac{\partial T}{\partial z}\Delta t.$$

Equation (15) is used to update the internal energy at the end of time increment U(t+Δt) using the energy at the beginning of the time increment U(t):

(16) U(t+Δt)=U(t)+ΔU.

In the case that the internal energy is a function of time, temperature, remaining mass, and their partial derivative with respect to dimensional coordinates; the total derivative of the internal energy with respect to time is showed as:

(17) $$\frac{dU}{dt}=\frac{\partial U}{\partial t}+\frac{\partial U}{\partial T}\frac{dT}{dt}+\frac{\partial U}{\partial \left(\partial T/\partial x_{i}\right)}\frac{d\left(\frac{\partial T}{\partial x_{i}}\right)}{dt}+\frac{\partial U}{\partial \rho }\frac{d\rho }{dt}+\frac{\partial U}{\partial \left(\partial \rho /\partial x_{i}\right)}\frac{d\left(\frac{\partial \rho }{\partial x_{i}}\right)}{dt}.$$

Compare terms in Equations (13) and (17), yielding:

(18) $$\frac{\partial U}{\partial T}=C_{p},$$

(19) $$\frac{\partial U}{\partial \left(\partial T/\partial x_{i}\right)}=0,i=1,2,3\;x_{1}=x,x_{2}=y,x_{3}=z.$$

Based on Equation (14), the partial derivative of heat flux vector with respect to temperature and temperature dimensional gradient can be presented by:

(20) $$\frac{\partial f}{\partial T}=-\frac{\partial T}{\partial x}\frac{\partial k_{1}}{\partial T}i-\frac{\partial T}{\partial y}\frac{\partial k_{2}}{\partial T}j-\frac{\partial T}{\partial z}\frac{\partial k_{3}}{\partial T}k,$$

(21) $$\frac{\partial f}{\partial \left(\partial T/\partial x_{i}\right)}=[\begin{array}{ccc} -k_{1} & 0 & 0\\ 0 & -k_{2} & 0\\ 0 & 0 & -k_{3} \end{array}],i=1,2,3\;x_{1}=x,x_{2}=y,x_{3}=z.$$

**Figure 3:**
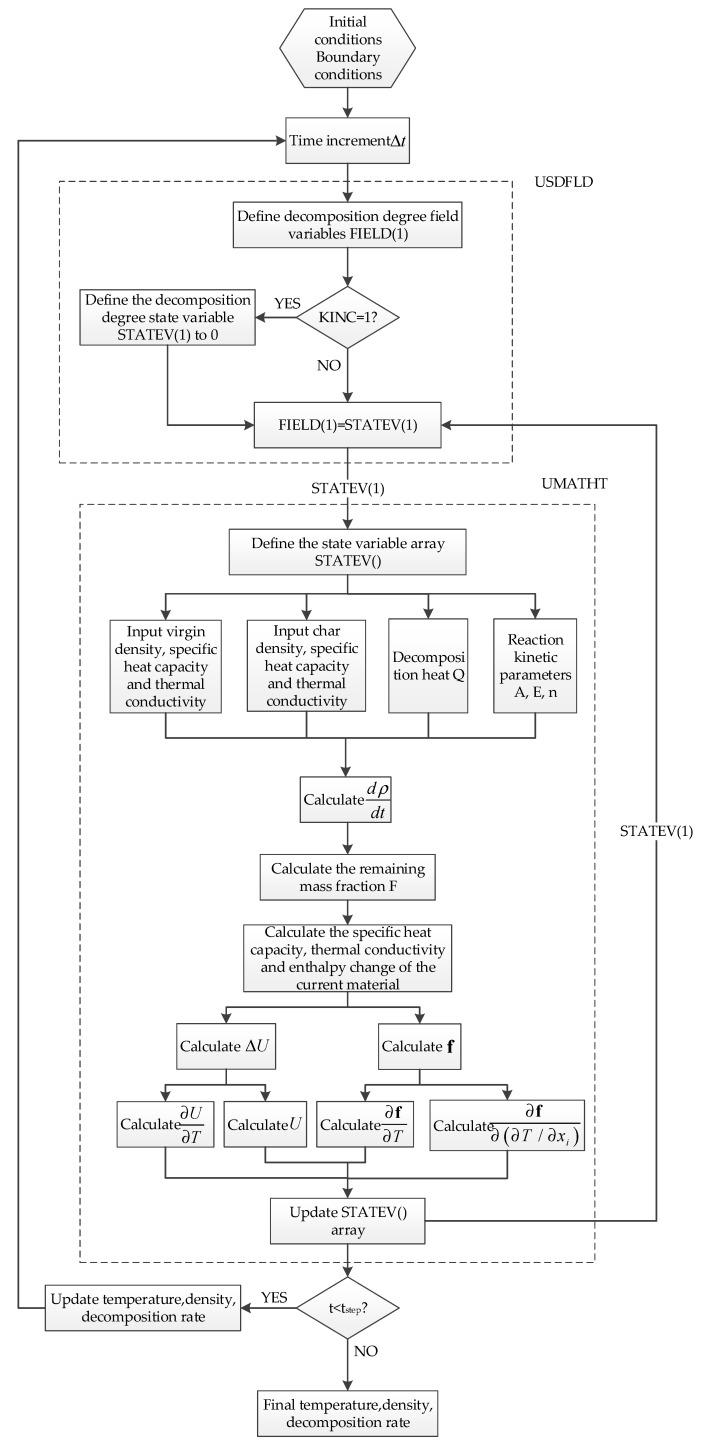
Flow chart of the thermal response calculation.

Figure 3 shows the flow chart of the calculation. When the resin matrix in the composite material does not decompose, the decomposition degree is 0; when the resin matrix is completely carbonized, the decomposition degree is 1. In the subroutine, the decomposition degree and decomposition rate of the material were obtained by a kinetic equation and stored in the state variable array STATEV(); the state variable STATEV(1) was updated and passed to the subroutine USDFLD to obtain the decomposition rate. The internal thermal energy of the material and the conducted heat flux were calculated by the UMATHT module. The changed heat was also transferred to the ABAQUS thermal analysis module to obtain an updated temperature field and decomposition rate field, which was continuously cycled and iterated by superimposing the time increment, until the end of the analysis step to get the final temperature field, decomposition rate field, and so on.

## 4. Results and Discussion

### 4.1. Field of Temperature

**Figure 4:**
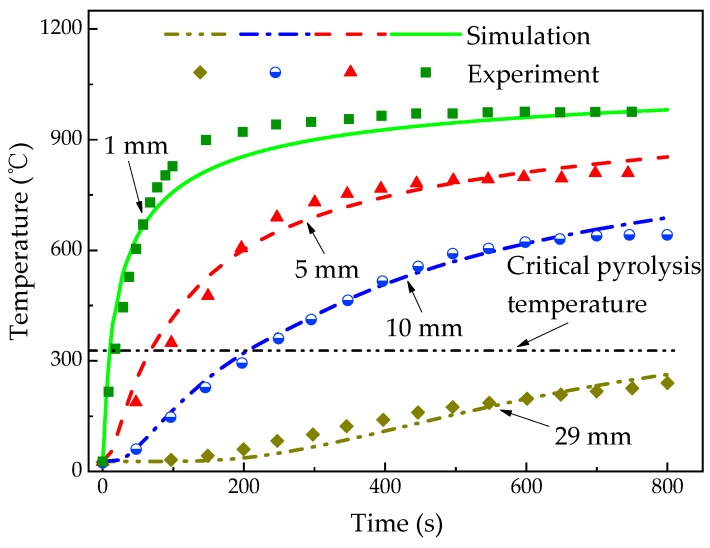
Temperature profiles of the glass fiber/phenolic resin composites under one-sided heating.

Figure 4 shows the temperature profiles of glass fiber/phenolic resin composites at *z* = 1, 5, 10, and 29 mm, respectively, which indicates a good agreement between calculated and experimental values. There is a rapid temperature increase at the 1 mm position at the initial heating stage, and then the temperature increase rate at *z* = 1 mm reduced obviously after 100 s, which is a typical nonlinear phenomenon. The leading reason is that the pyrolysis reaction occurs, which absorbs the heat to some extent, altering the rate of temperature increase. Moreover, the temperature increase of the exposed surface leads to the enhancement of heat dissipation capacity of the sample. In addition, the diffusion of pyrolysis gas takes part of the heat to the surrounding. To sum up, these reasons result in a reduction in net heat flux of exposed surface.

It is necessarily noted that the calculated data at *z* = 1 mm is slightly below the experimental data with time increase. The model did not take into account the thermal expansion, internal pressure change, and the heat transfer between the pyrolysis gas and the solid material. When comparing with the results by Li [46], the accuracy of the predicted temperature is improved by 22%. From the results, the temperature gradually increased with the heating time in *z* = 5 and 10 mm position. However, there is no significant temperature change at *z* = 29 mm within 200 s. At the later stage of the test, the temperature at *z* = 1 mm is almost steady. The calculated value and the test data are consistent. At 800 s, the temperatures at *z* = 1, 5, and 10 mm reached 981 °C, 853 °C, and 688 °C, respectively, which all exceeded the critical pyrolysis temperature, while the 262 °C at *z* = 29 mm did not reach the critical pyrolysis temperature, which means that the material did not decompose.

**Figure 5:**
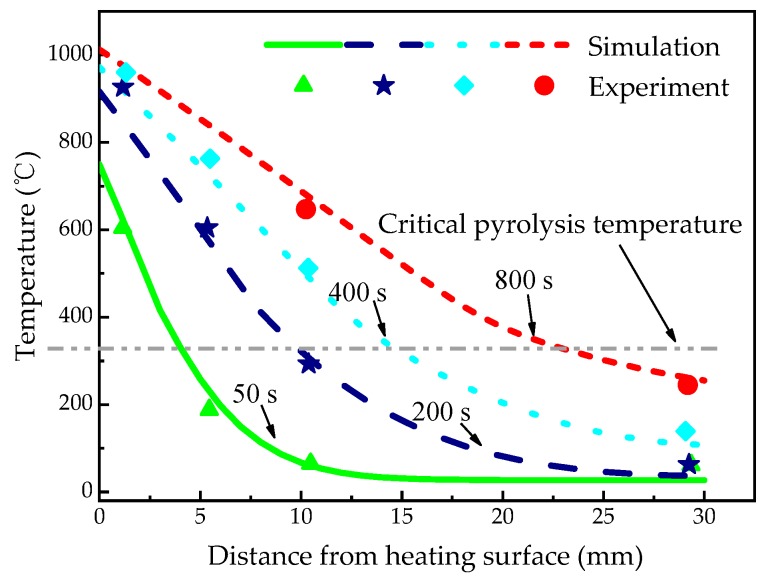
Spatially dependent temperature profiles of the glass fiber/phenolic resin composites specimen under one-sided heating.

Figure 5 shows the temperature distribution in the sample at 50, 200, 400, and 800 s, respectively. The results also indicate a good agreement between the calculated and experimental temperature. However, the variation of temperature distribution with the sample length and heating time is not mentioned in reference [8]. The temperature gradient changed with the increasing time and gradually tended to be linear from nonlinearity. It is also shown that the temperature gradient of the sample decreased with the increase of the depth at different time. Specially, the temperature curve at 50 s represents a strong nonlinear distribution at the early stage of test, while that at 800 s is almost linear.

**Figure 6:**
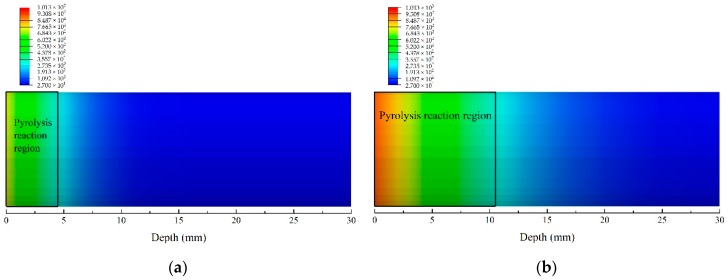

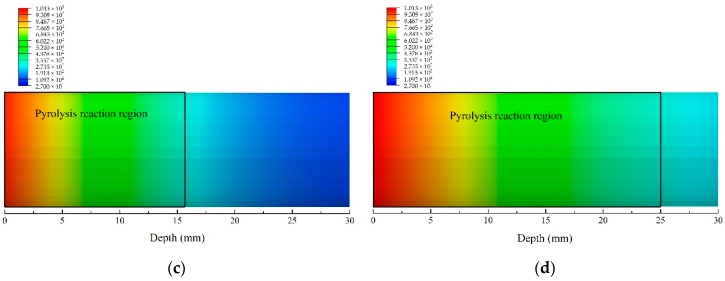
Temperature contours of the glass fiber/phenolic resin composites at different times under one sided heating. (**a**) 50 s; (**b**) 200 s; (**c**) 400 s; (**d**) 800 s.

Figure 6 shows the temperature contour of FE model at 50, 200, 400, and 800 s, respectively. Pyrolysis reaction region, which is defined using pyrolysis temperatures in this work, should be identified before analysis of decomposition process. At 50 s, pyrolysis reaction has begun to occur, and the depth of the sample affected by pyrolysis reaction was 4.51 mm. Furthermore, the energy transferred to the back of the material in the length direction gradually increases with the heating time, which results in the temperature increase of the whole sample and the enlargement of the region where pyrolysis reaction is in progress in the length direction. At 200 s and 400 s, the range of the region where the pyrolysis reaction is in progress were 10.5 mm and 15.65 mm, respectively. At 800 s, the material within the range of 1/6 depth close to the back did not reach the critical pyrolysis temperature, which was still the virgin material. However, the pyrolysis reaction will stop when the matrix is decomposed thoroughly, resulting in the invalidity of pyrolysis reaction region defined using pyrolysis temperatures. Therefore, char region [43] identified in this work is defined according to the char density, discussed in the following sections.

### 4.2. Density and Decomposition Degree

**Figure 7:**
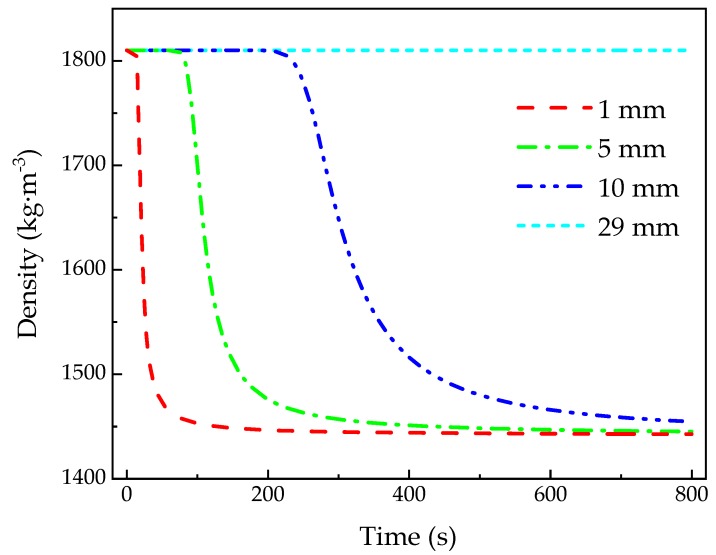
Density curves of the glass fiber/phenolic resin composites under one-sided heating.

Figure 7 shows density–time curves calculated at *z* = 1, 5, 10, and 29 mm, respectively, which is often difficult to measure and verify experimentally. At 17 s, the material at *z* = 1 mm undergone thermal decomposition and the density reached the char density in a short time, while the pyrolysis reaction that materials at *z* = 5 and 10 mm undergo began at 72 s and 203 s, respectively. In addition, the material at *z* = 29 mm did not reach the critical pyrolysis temperature, and still remain virgin density during the test. All in all, the time to reach the critical pyrolysis temperature increases with the depth, in addition to the time to occur the pyrolysis reaction. On the other hand, the time that the density changed from virgin to char at *z* = 1, 5, and 10 mm was 61 s, 215 s, and 505 s, respectively. It can be implied that, as the depth increases, the longer the material takes to complete the pyrolysis reaction, and the slower the pyrolysis process becomes. This phenomenon is consistent with the description of previous paragraph and is attributed to two reasons. First, the pyrolysis reaction of phenolic resin occurs in a certain temperature range, 300–900 °C indicated in reference [8]. Second, the closer the material is to the heated surface, the higher the temperature increase rate becomes, which results in the reduction of the pyrolysis reaction time.

**Figure 8:**
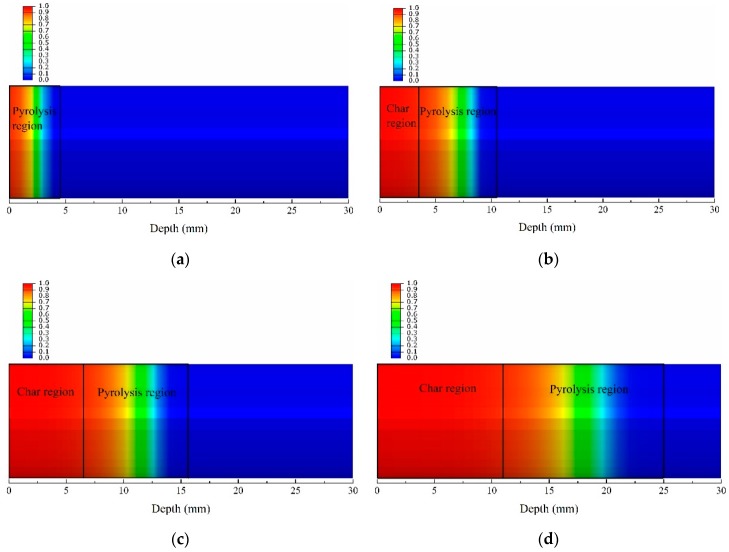
Decomposition degree contours of the glass fiber/phenolic resin composites at different times under one-sided heating. (**a**) 50 s; (**b**) 200 s; (**c**) 400 s; (**d**) 800 s.

Figure 8 shows the decomposition degree contours of the glass fiber phenolic resin composite, which equals one minus F, which is defined in Equation (4). In fact, decomposition degree is the expression of dimensionless treatment of density, which is usually used for relative research [29,47,48]. Namely, the decomposition degree of 0 represents the fact that the material density is the virgin density, and the decomposition degree of 1 represents that the material density is the char density. At 50 s, the material close to the heated surface has not completely decomposed, and the range in the pyrolysis region was 4.50 mm. With increasing heating time, the char region began to appear. The range of the char region were 3.56 mm and 6.49 mm, at 200 s, and 400 s, respectively, while the range of region where the pyrolysis reaction was undergoing were 6.94 mm and 9.16 mm, respectively. At 800 s, the range of the material char region and the pyrolysis region were 10.97 mm and 14.03 mm, respectively. It is noted that the char region and the pyrolysis region enlarged further with increasing time. This may be due to the increasing thermal conductivity caused by the temperature increase, resulting in the heat transfer being faster. Thus, the char region and pyrolysis region can be distinguished definitely, which is an appropriate metric for predicting the likelihood of cracking or delamination caused by decomposition, for helping to evaluate the thermal behavior of composites [30].

**Figure 9:**
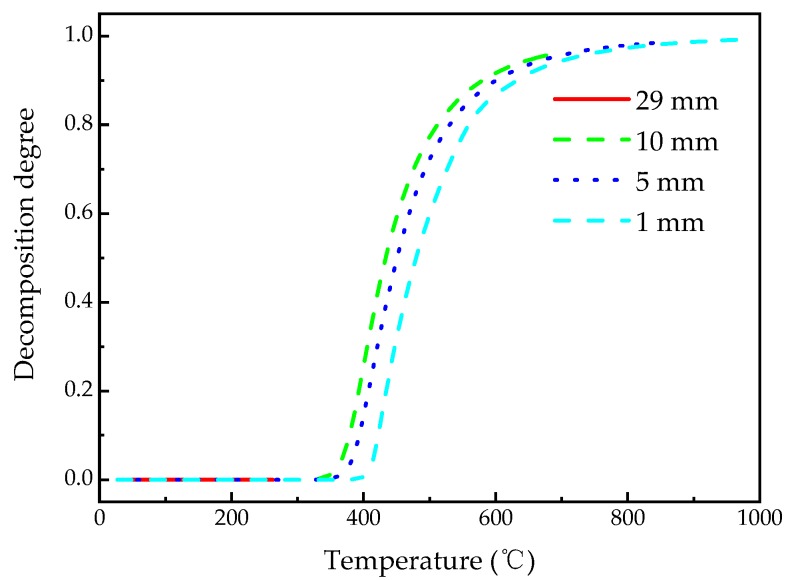
The change of decomposition degree with temperature for different positions of the glass fiber/phenolic resin composites.

Figure 9 shows the decomposition degree–temperature curves calculated at *z* = 1, 5, 10, 29 mm, which is similar to the typical TGA curves of polymer material at different rates of temperature increase. There is a phenomenon neglected easily by researchers [8,30] in calculations that the temperatures when the char density is reached at different depths are different. The materials at *z* = 1, 5, 10 mm reached the char density at 714 °C, 682 °C, and 659 °C, respectively. Furthermore, when the same decomposition degree is achieved at different depths, the material temperatures are different. Namely, the further the depth position from the heating surface is, the lower the temperature of the material at this location. On the other hand, when the temperatures at different depths are the same, their decomposition degrees are different. Namely, at a certain temperature, the closer the position is to the heated surface, the lower the decomposition degree of the material at this position. This is because, when the same temperature or decomposition degree of material is achieved, the rates of temperature increase at different depths are different, while the pyrolysis reaction of phenolic resin is affected by the rate of temperature increase [49]. The smaller the rate of temperature increase, the more adequate the pyrolysis reaction of phenolic resin.

### 4.3. Decomposition Rate

**Figure 10:**
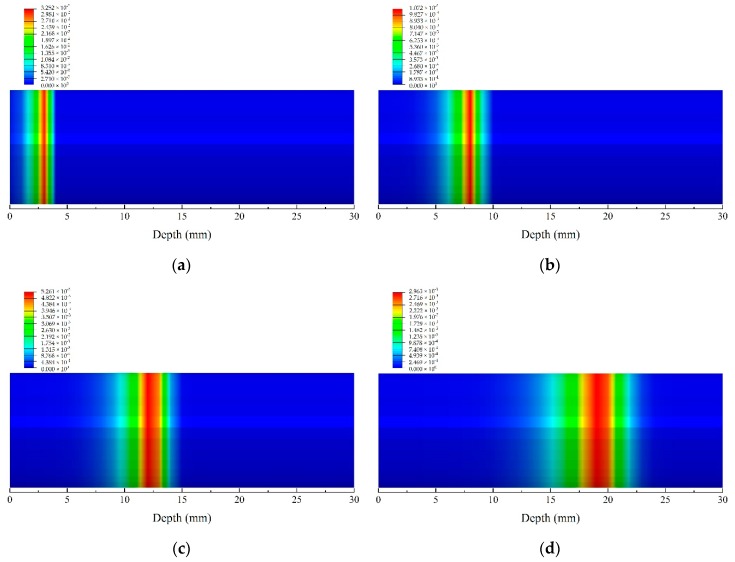
Decomposition rate contours of the glass fiber/phenolic resin composites specimen at different times under one-sided heating. (**a**) 50 s (**b**); 200 s; (**c**) 400 s; (**d**) 800 s.

Figure 10 shows the contours of the decomposition rate of glass fiber/phenolic resin composites at 50, 200, 400, and 800 s, respectively. Specifically, the higher the decomposition rate is, the greater the change of the decomposition degree per unit of time is, indicating that the more intense the material pyrolysis reaction is [34]. At 50 s, the peak of decomposition rate, which occurred at *z* = 3.02 mm, is 0.034 s^−1^, indicating the location of the most intense pyrolysis reaction at this time. In addition, the location of peak of decomposition rate at 200 s and 400 s were 8.01 mm and 12.00 mm, respectively. Therefore, this location, representing the decomposition front, moves towards the back of the material with the increasing time. Furthermore, the maximum decomposition rate of the material gradually decreases with the increase of time. At 800 s, the material decomposition rate reaches the peak of 0.003 s^−1^, which is located at a depth of 19.03 mm.

**Figure 11:**
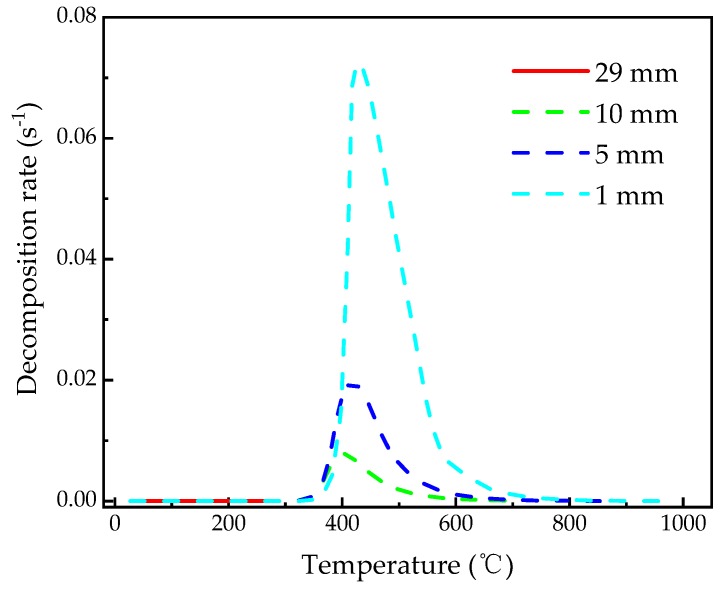
The change of decomposition rate with temperature for different positions of the glass fiber/phenolic resin composites.

Figure 11 shows the decomposition rate-temperature curves calculated at *z* = 1, 5, 10, 29 mm, respectively. It is similar to the typical DTG curves of polymer material at different rates of temperature increase [49], which is usually obtained from TGA and difficult to be derived in the practical coupons, elements and components. This capability in FE simulation is beneficial for comprehension on the decomposition process. The respective peaks of decomposition rate of the glass fiber/phenolic resin composites at *z* = 1, 5, and 10 mm were 0.072 s^−1^, 0.02 s^−1^. 0.0078 s^−1^, which were achieved at temperature of 425 °C, 418 °C, and 402 °C, respectively. It is concluded that the farther the position from the heated surface is, the smaller the peak of the decomposition rate at this position is, and the lower the temperature at which the peak of the decomposition rate is reached. The reason is that the temperature increase rates are different when the peaks of the decomposition rates at different depth are reached. The closer the depth position from the heated surface is, the higher the temperature increase rate at this location, which causes the maximum decomposition rate of the composite shifts to the region of higher temperatures. Additionally, it is noted that the temperature range of the pyrolysis reaction broadens with closer distance from the heated surface.

### 4.4. Thermophysical Properties

**Figure 12:**
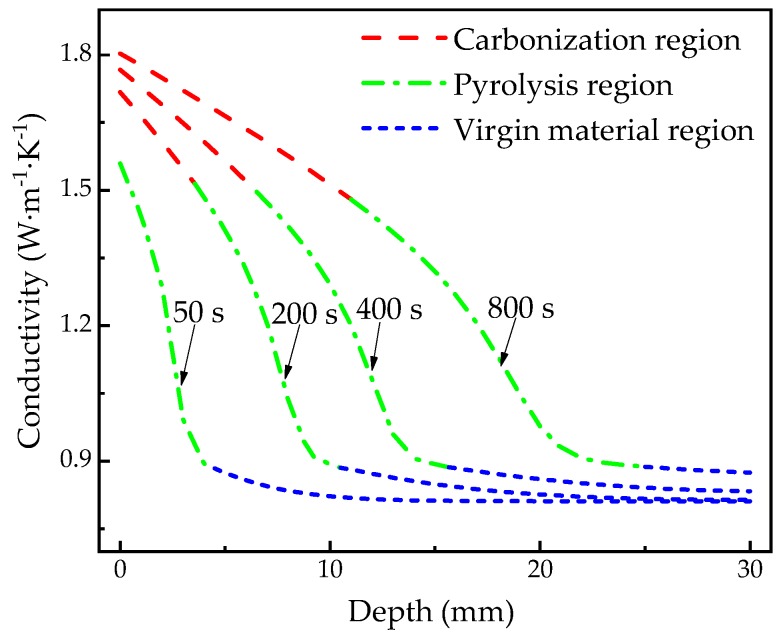
Thermal conductivity with depths at 50, 200, 400, 800 s.

**Figure 13:**
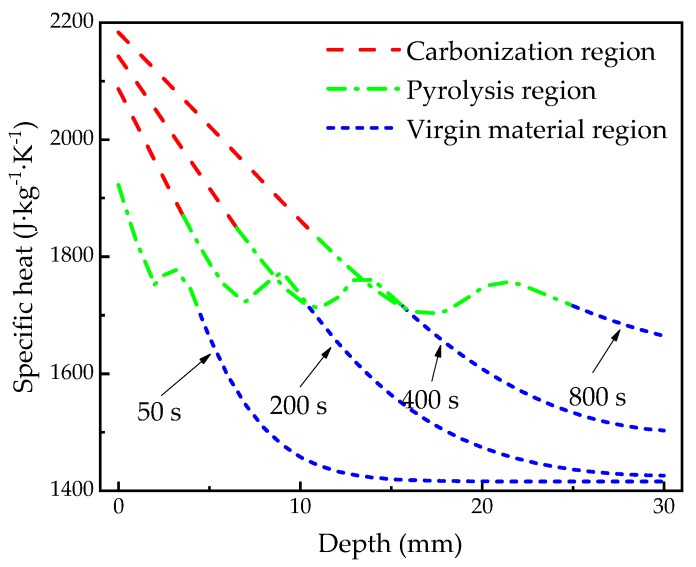
Specific heat capacity with depths at 50, 200, 400, 800 s.

Figure 12 shows the spatial-dependent thermal conductivity calculated at 50, 200, 400, 800 s, respectively, which is often difficult to measure and verify experimentally, but can help with interpreting some phenomenon and mechanism of heat transfer or even decomposition. It is shown that the thermal conductivity of the carbonization region and the pyrolysis region gradually decreases with depths. However, that of the virgin material region does not change much as the depth increases. The reason is that the temperature gradient of the virgin material area is relatively less than the other two regions as shown in Figure 5. Figure 13 shows the spatial-dependent specific heat capacity calculated at 50, 200, 400, 800 s, respectively. Similar to the spatial-dependent thermal conductivity, the specific heat capacity of the carbonization region and the virgin material region gradually increases with the heating time, and gradually decreases with the increase of depth. Although the specific heat capacity of the material in the pyrolysis region decreases generally along the depth, there is a fluctuation of specific heat capacity with the increase of the depth. This may be due to the fact that the specific heat capacity of the material in the pyrolysis zone is not only affected by temperature, but also related to the degree of pyrolysis of the material, indicating the complexity of the decomposition of materials.

## 5. Conclusions

Taking into account heat conduction, matrix pyrolysis, and gas diffusion, a transient three-dimensional numerical model for glass fiber/phenolic composite under a one-sided heat flux has been established via ABAQUS code to predict and analyze the thermal response of material. In that, the process of matrix pyrolysis and pyrolysis gas diffusion was implemented in the UMATHT and USDFLD subroutines. 

By comparing the results simulated using the method proposed in this paper with experimental data and the calculated results by reference, the accuracy of the predicted temperature profiles can be proved, indicating that the model and method developed can capture the nonlinear thermal behavior of the glass fiber/phenolic composite. Some characteristics that are difficult to measure in the experiments, such as density at a certain location with time, decomposition degree at a certain location with temperature, decomposition rate with temperature, and thermophysical properties at different locations with time, can be extracted in the simulation to be used to evaluate the thermal behavior, such as the process of decomposition and carbonization.

Some regularities of the decomposition of glass fiber/phenolic composite were found out. For example, as the depth increases, the longer the material takes to complete the pyrolysis reaction, and the slower the pyrolysis process becomes. In addition, at a certain temperature, the closer the position from the heated surface is, the lower the decomposition degree of the material at this position is. Furthermore, the farther the position from the heated surface is, the smaller the peak of the decomposition rate at this position, and the lower the temperature at which the peak of the decomposition rate is reached.

It should be pointed out that thermal behavior in the macro-scale was correlated with the decomposition features and thermophysical properties of glass fiber/phenolic composite via the FE simulation developed in this work, and char region and pyrolysis region can be identified definitely using pyrolysis temperature and char density in the FE simulation, which maybe were valuable for the prediction and comprehension of thermal behavior of glass fiber/phenolic composites. In addition, it can provide an effective numerical method for thermal protection design of composite structure. In future, the structural effect on the heat transfer and decomposition in primary structures and interior structure of aircraft manufactured by composites can be studied.

## Figures and Tables

**Table 1:** Material performance for glass fiber/phenolic composite.

Parameter	Value
Resin volume fraction	0.395
Fiber volume fraction	0.605
Virgin density/kg·m^−3^	1810
Final char density/ kg·m^−3^	1440
Virgin thermal conductivity/ W·m^−1^·K^−1^	0.804 + 2.76 × 10^−4^ *T*
Final char thermal conductivity/ W·m^−1^·K^−1^	0.955 + 8.42 × 10^−4^ *T*
Virgin specific heat/ kJ·kg ^−1^·K^−1^	1.089 + 1.09 × 10^−3^ *T*
Final char specific heat/ kJ·kg ^−1^·K^−1^	0.870 + 1.02 × 10^−3^ *T*
Specific heat of gases/ kJ·kg ^−1^·K^−1^	9.63
Activation energy/ J·mol^−1^	2.60 × 10^5^
Reaction order	6.3
Pre-exponential factor/ s^−1^	8.16 × 10^18^
Decomposition heat/ kJ·kg^−1^	234.0

Note: *T*—Temperature.
